# Determination of Factors Affecting Dengue Occurrence in Representative Areas of China: A Principal Component Regression Analysis

**DOI:** 10.3389/fpubh.2020.603872

**Published:** 2021-01-18

**Authors:** Xiaobo Liu, Keke Liu, Yujuan Yue, Haixia Wu, Shu Yang, Yuhong Guo, Dongsheng Ren, Ning Zhao, Jun Yang, Qiyong Liu

**Affiliations:** ^1^State Key Laboratory of Infectious Disease Prevention and Control, Collaborative Innovation Center for Diagnosis and Treatment of Infectious Diseases, WHO Collaborating Centre for Vector Surveillance and Management, National Institute for Communicable Disease Control and Prevention, Chinese Center for Disease Control and Prevention, Beijing, China; ^2^Provincial Hospital Affiliated to Shandong First Medical University, Jinan, China; ^3^The Collaboration Unit for Field Epidemiology of State Key Laboratory of Infectious Disease Prevention and Control, Nanchang Center for Disease Control and Prevention, Nanchang, China; ^4^Institute for Environmental and Climate Research, Jinan University, Guangzhou, China

**Keywords:** dengue, influencing factors, principal component analysis, mosquito-borne disease, control

## Abstract

**Background:** Determination of the key factors affecting dengue occurrence is of significant importance for the successful response to its outbreak. Yunnan and Guangdong Provinces in China are hotspots of dengue outbreak during recent years. However, few studies focused on the drive of multi-dimensional factors on dengue occurrence failing to consider the possible multicollinearity of the studied factors, which may bias the results.

**Methods:** In this study, multiple linear regression analysis was utilized to explore the effect of multicollinearity among dengue occurrences and related natural and social factors. A principal component regression (PCR) analysis was utilized to determine the key dengue-driven factors in Guangzhou city of Guangdong Province and Xishuangbanna prefecture of Yunnan Province, respectively.

**Results:** The effect of multicollinearity existed in both Guangzhou city and Xishuangbanna prefecture, respectively. PCR model revealed that the top three contributing factors to dengue occurrence in Guangzhou were Breteau Index (BI) (positive correlation), the number of imported dengue cases lagged by 1 month (positive correlation), and monthly average of maximum temperature lagged by 1 month (negative correlation). In contrast, the top three factors contributing to dengue occurrence in Xishuangbanna included monthly average of minimum temperature lagged by 1 month (positive correlation), monthly average of maximum temperature (positive correlation), monthly average of relative humidity (positive correlation), respectively.

**Conclusion:** Meteorological factors presented stronger impacts on dengue occurrence in Xishuangbanna, Yunnan, while BI and the number of imported cases lagged by 1 month played important roles on dengue transmission in Guangzhou, Guangdong. Our findings could help to facilitate the formulation of tailored dengue response mechanism in representative areas of China in the future.

## Introduction

Dengue fever is a mosquito-borne viral infection causing severe flu-like illness, and sometimes causing a potentially lethal complication called dengue shock syndrome. Globally, the incidence of dengue has increased 30-fold over the past 50 years (1). Driven by climatic and environmental changes (2), globalization, urbanization (3, 4), vector activity, and human behavior change (5), or the combination of these favorable conditions (6, 7), dengue outbreaks have been continuously occurring in southern China such as Guangdong (8–13) and Yunnan (14–17) since 2013, which has posed an important public health threat in China (18–23).

Assessing the impact of some driving factors such as vectors and climate-related features on the occurrence of dengue has become one of hotspots in current research (24–28). For instance, some studies have attempted to explore the influencing factors of dengue occurrence in some representative regions such as Guangdong and Yunnan provinces, and reported the potential dengue-related factors, including temperature, relative humidity, precipitation, sunlight and mosquito density (29–31). However, considering the strong correlations among these multi-dimensional factors, the effect of multicollinearity among independent variables has not been well examined in most previous studies, which could severely distort the model estimation. Principal component regression (PCR) can effectively minimize the multi-collinearity among the factors and has been widely used in the field of biomedicine (32, 33). The pathogenesis of dengue is complex, involving viruses, hosts, human populations, ecological, environmental and social factors and the interactions of these factors. It is therefore necessary to take into account many factors in the model estimation and meanwhile to minimize the possible impact from multicollinearity.

In view of this, this study selected two representative cities or prefectures from two provinces with frequent dengue outbreak in recent years having different ecological, meteorological and socioeconomic characteristics, and then established the PCR model to identify the key factors influencing the occurrence of dengue. The results may provide scientific evidence for the formulation of tailored dengue prevention and control strategies and measures in China in the future.

## Methodology

### Research Site

**Figure 1:**
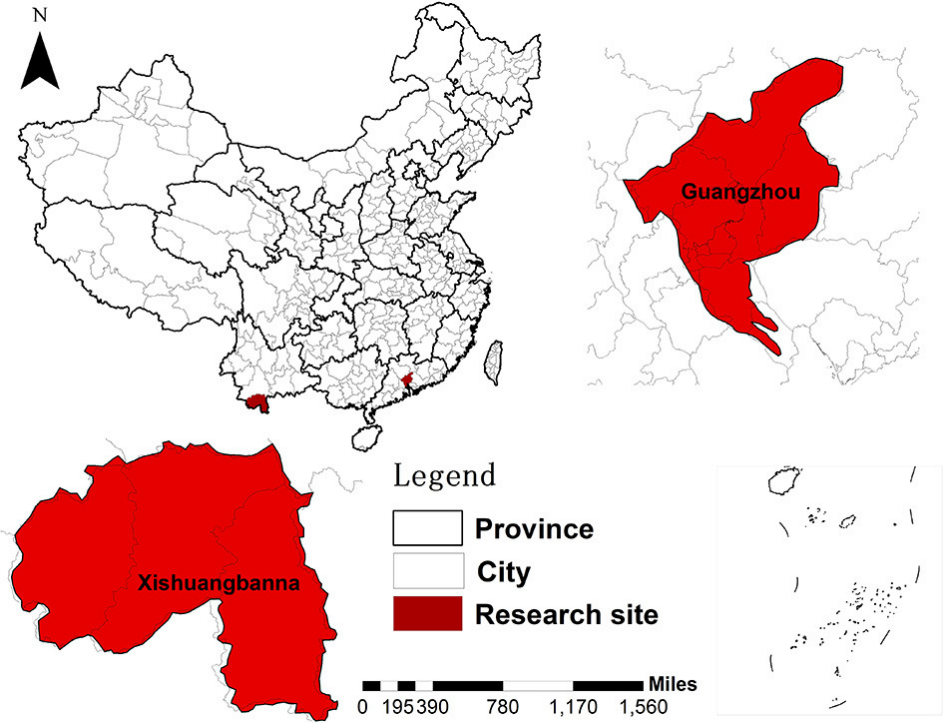
The research sites of the current research: Xishuangbanna prefecture in Yunnan province and Guangzhou city in Guangdong province.

In this study, Xishuangbanna Dai Autonomous Prefecture (hereinafter referred to as Xishuangbanna prefecture) in Yunnan Province and Guangzhou city in Guangdong Province were selected as two representative sites with high-risk of dengue in China based on epidemic situation of dengue in during recent years (Figure 1).

Xishuangbanna prefecture is located at 21°10′ to 22°40′ north latitude, 99° 55′ to 101°50′ east longitude. It belongs to the humid tropical area in south of the equator and connects Jiangcheng county and Puer city in the East and West. It is adjacent to Lancang county in the northwest, Laos and Myanmar in the southeast, south and southwest, respectively. Covering an area of 19, 582.45 square kilometers, there were 1.196 million permanent residents in this prefecture in 2019.

Guangzhou, the capital city of Guangdong Province, also known as Sub-provincial City, National Central City and Mega-city, is located at 22° 26′ to 23° 56′ north latitude, 112° 57′ to 114° 3′ east longitude. It situates on a slope terrain with higher altitude in the northeast and lower altitude in the southwest. The northern part of the city is a hilly and mountainous area with concentrated forests. Guangzhou is adjacent to the subtropical coast and the equator passes through the south-central part of this metropolis, one of the largest cities in China. A marine subtropical monsoon climate prevails in this area, which is characterized by warm and rainy long summer, abundant light and heat, and a short frost period in winter. The annual average temperature ranges 20–22°C with the smallest annual average temperature difference. Covering an area of 7,434.4 square kilometers, there were 15.359 million permanent residents in this city in 2019.

### Data Collection

Records of dengue cases, the number of imported dengue case, the number of imported dengue case lagged by 1 month [Im1] from Guangzhou city and Xishuangbanna prefecture between 2006 and 2017 were obtained from China Notifiable Infectious Disease Reporting System. All dengue cases were diagnosed according to the China National Diagnostic Criteria for dengue (WS216-2008). Information of dengue cases included gender, age, occupation, date of onset, clinical diagnosis, or laboratory confirmed cases, indigenous or imported cases. The criteria of imported or indigenous cases are as described in previous study (34).

Breteau Index (BI) is an index for surveillance *Aedes* larvae density and can be calculated by number of positive containers per a hundred houses inspected. At present, it is utilized widely in dengue risk assessment. The BI of high-risk areas in Guangzhou city and Xishuangbanna prefecture from June to October from 2006 to 2017 was obtained from the annual surveillance report of major infectious diseases and vectors in China CDC.

According to the major meteorological factors affecting dengue occurrence in representative areas of China in the previous researches, and also considering the availability of data of different indicators at different time scales, monthly meteorological data from 2006 to 2017 were obtained from the National Meteorological Science Data Sharing Service (http://cdc.nmic.cn/home.do). These indicators includes monthly average of temperature (Tmean), monthly average of temperature lagged by 1 month (Tmean1), monthly average of maximum temperature (Tmax), monthly average of maximum temperature lagged by 1 month (Tmax1), monthly average of minimum temperature (Tmin), monthly average of minimum temperature lagged by 1 month (Tmin1), monthly average of relative humidity (Hum%), cumulative precipitation (CP), cumulative precipitation lagged by 1 month (CP1), days of precipitation (DP) in the current month, days of precipitation lagged by 1 month (DP1).

The population data over the study period were retrieved from the Guangdong and Yunnan Statistical Yearbooks. The collected dataset of dengue indigenous and imported cases, the key meteorological index and BI data from June to October during 2006–2017 were integrated for the subsequent principal component regression (PCR) analysis.

### Statistical Analysis

Multiple linear regression analysis was firstly utilized to determine the possible multiple collinearities of different variables included in this study. Collinearity diagnostic was carried out, and eigenvalue and condition index were computed. All the analyses were analyzed by SPSS version 18.0 (SPSS Inc., Chicago, IL, USA).

The variables concerning dengue occurrence in this study were standardized at first using normalization method. Principal component analysis was adopted to determine the number of principal components of standardized key factors including meteorological factors, mosquito density and imported dengue cases in the last month in the selected regions of Guangdong and Yunnan Provinces in China.

Taking the number of dengue cases as dependent variable (y) and principal components as independent variable (x), we then adopted the principal components regression analysis. In detail, according to the determined principal component, the principal component in front order replaces the original independent variable (x) for multiple linear regression analysis to obtain the regression model between the standardized independent variable (x) and the dependent variable (y). The number of principal components was determined by the accumulative contribution rate of these principal components by total initial eigenvalues. Then, the standardized independent variable is reduced to the original independent variables to obtain the regression model of the original independent variable and the independent variable. In this study, the established models are as follows.

- The structure of established principal component regression model using standardized scores of principal components
  Y = b_0_+b_1_Z_1_+b_2_Z_2_+b_3_Z_3_+……b_p_Z_p_, where b_1_, b_2_,…, b_p_ are called as principal component contribution rate.
- The structure of established regression model using original variable:
  Y = A+β_1_X_1_+β_2_X_2_+β_3_X_3_+……β_p_X_p_, where β_1_, β_2_,…, β_p_ are called as marginal coefficient.

## Results

### General Situation of Variables in the Research Site From 2006 to 2017

Table 1 presents the general composition of dengue and the influencing factors in Guangzhou city of Guangdong province and Xishuangbanna prefecture of Yunnan province, from 2006 to 2017. Totally, 60 records in Guangzhou city and 60 records in Xishuangbanna prefecture were included in the current analysis. And the minimum value, the maximum value, mean and standard deviation were included.

**Table 1:** Comparison of the dengue and the influencing factors in two research sites, 2006–2017.

**Research sites**	**Variable**	***N***	**Minimum value**	**Maximum value**	**Mean**	**Standard deviation**
Guangzhou city, Guangdong	Im	60	0	19790	713.30	3346.26
	Im1	60	0	59	3.95	8.64
	BI	60	2	13	5.33	1.91
	Tmean (°C)	60	23	31	27.46	1.86
	Tmean1 (°C)	60	25	31	27.71	1.38
	Hum (%)	60	58	87	77.26	6.35
	Tmin (°C)	60	19	28	24.34	1.95
	Tmin1 (°C)	60	22	28	24.68	1.29
	Tmax (°C)	60	28	35	32.11	1.71
	Tmax1 (°C)	60	29	35	32.19	1.57
	CP (mm)	60	1	835	233.62	158.47
	CP1 (mm)	60	37	835	299.43	165.62
	DP (d)	60	2	24	14.03	5.85
	DP1 (d)	60	7	25	16.48	4.50
Xishuangbanna prefecture, Yunnan	Im	60	0	672	55.57	151.94
	Im1	60	0	41	2.65	7.04
	BI	60	0	52	10.66	10.94
	Tmean (°C)	60	22	26	24.73	1.03
	Tmean1 (°C)	60	24	27	25.15	0.57
	Hum (%)	60	77	91	83.62	3.42
	Tmin (°C)	60	19	23	21.91	1.10
	Tmin1 (°C)	60	20	23	22.10	0.76
	Tmax (°C)	60	28	33	30.68	0.95
	Tmax1(°C)	60	29	34	31.24	1.025
	CP (mm)	60	23.6	555.9	222.40	128.98
	CP1(mm)	60	48	556	238.82	113.69
	DP (d)	60	5	28	18.75	5.97
	DP1 (d)	60	11	28	20.03	4.45

*Im means the number of dengue case; Im1 means the number of imported dengue cases lagged by 1 month; BI means Breteau index; Tmean means monthly average of temperature (°C); Tmean1 means monthly average of temperature lagged by 1 month (°C); Hum means monthly average of relative humidity (%); Tmin means monthly average of minimum temperature (°C); Tmin1 means monthly average of minimum temperature lagged by 1 month (°C); Tmax means monthly average of maximum temperature (°C); Tmax1 means monthly average of maximum temperature lagged by 1 month (°C); CP means the cumulative precipitation; CP1 means cumulative precipitation lagged by 1 month; DP means days of precipitation; DP1 means days of precipitation lagged by 1 month*.

**Figure 2:**
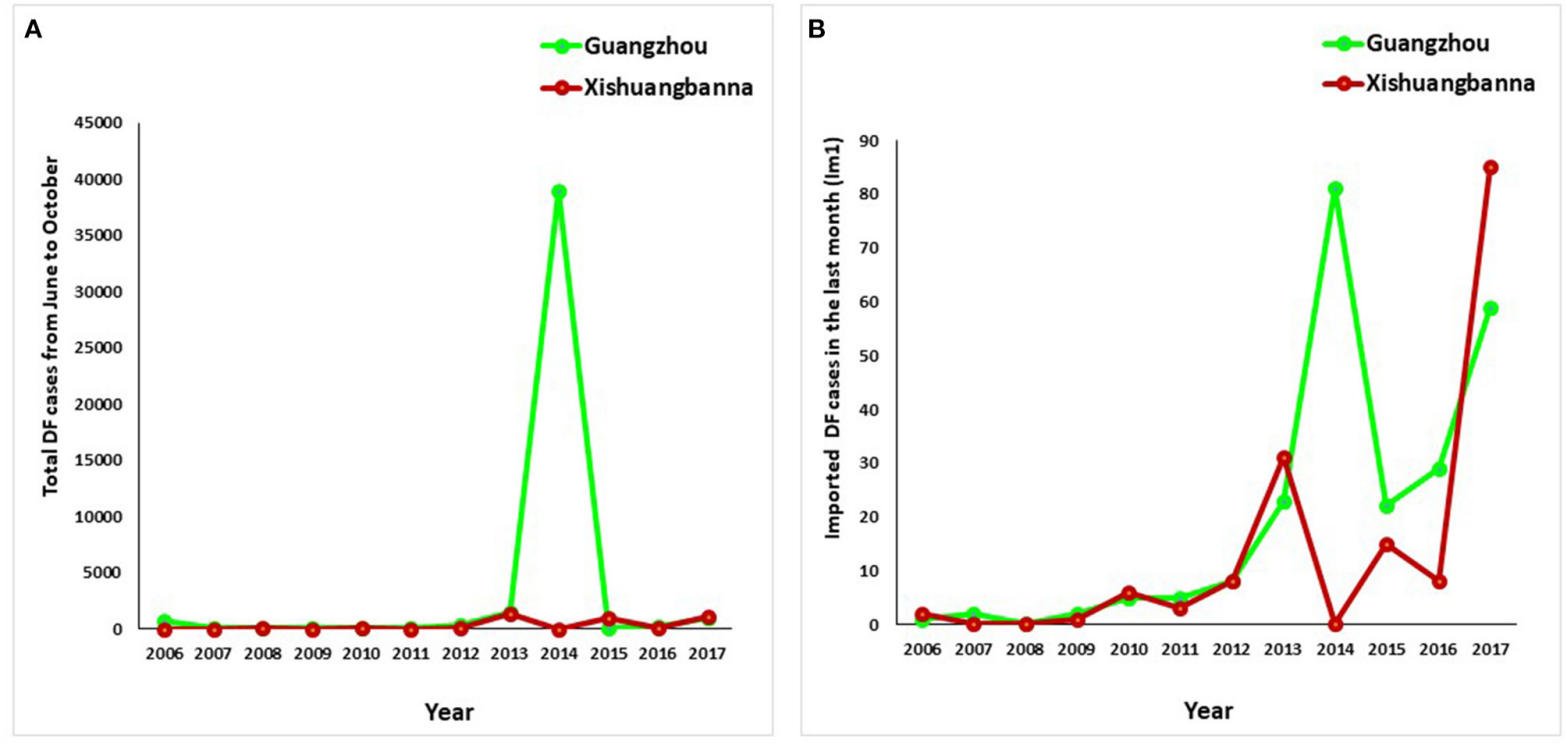
Temporal dynamics of dengue in the current month **(A)** and imported cases at last month **(B)** from June to October in the representative areas of China, 2006-2017.

Since 2013, the number of dengue cases have increased in fluctuation, with the highest in 2014 in Guangzhou (Figure 2A). For the imported dengue cases in prior month, it increased after 2009 and reached the highest in Guangzhou city in 2014 and in Xishuangbanna prefecture in 2017 (Figure 2B).

**Figure 3:**
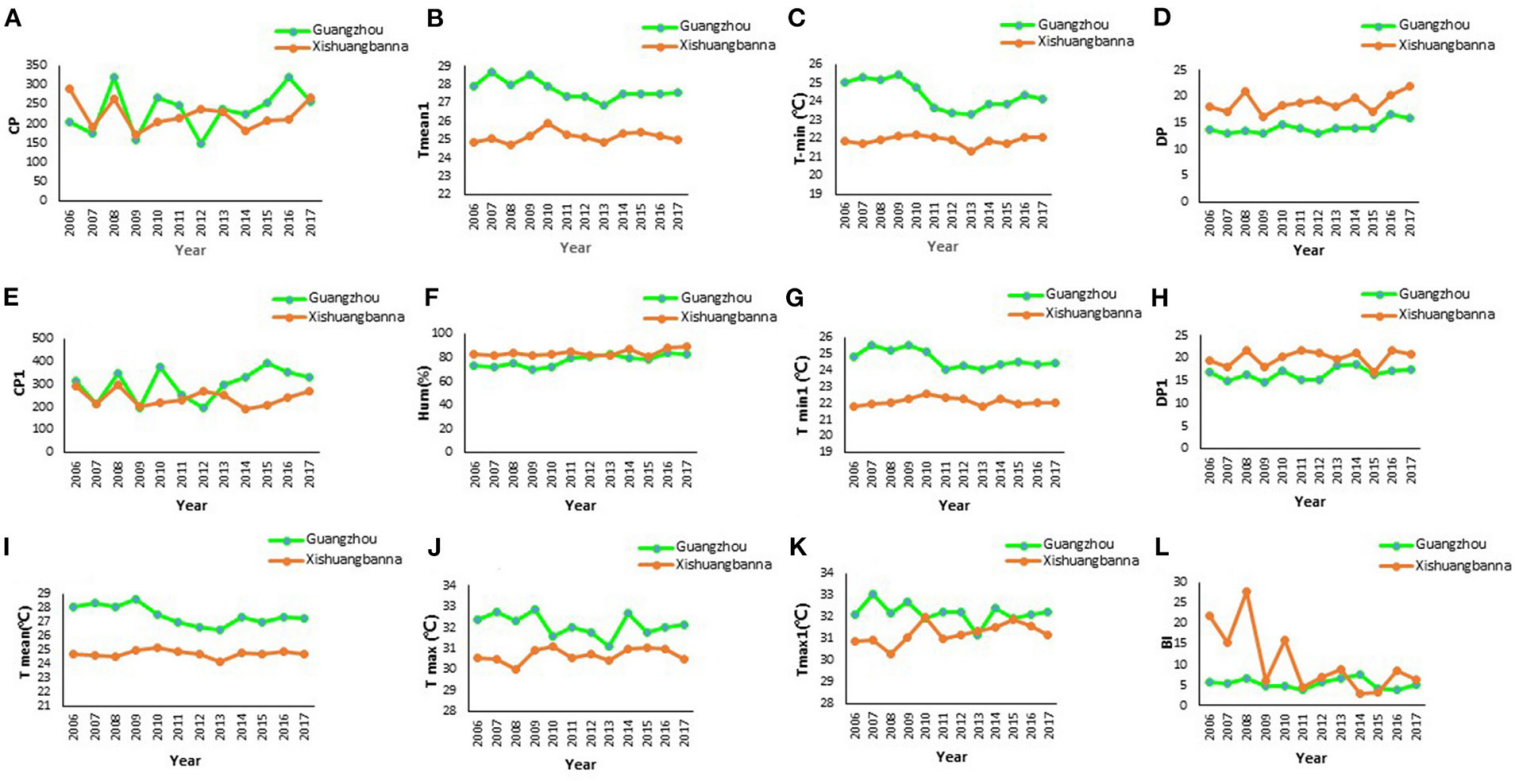
Temporal dynamics of some major variables in Guangzhou and Xishuangbanna, 2006–2017. **(A)** The cumulative precipitation (CP). **(B)** Monthly average of temperature lagged by 1 month (Tmean1). **(C)** Monthly average of minimum temperature (Tmin) (°C). **(D)** Days of precipitation (DP). **(E)** Cumulative precipitation lagged by 1 month (CP1). **(F)** Monthly average of relative humidity (Hum) (%). **(G)** Monthly average of minimum temperature lagged by 1 month (Tmin1) (°C). **(H)** Days of precipitation lagged by 1 month (DP1). **(I)** Monthly average of temperature (Tmean) (°C). **(J)** Monthly average of maximum temperature (Tmax) (°C). **(K)** Monthly average of maximum temperature lagged by 1 month (Tmax1) (°C). **(L)** Breteau Index (BI).

Figure 3 shows the temporal dynamics of the major factors in Guangzhou city and Xishuangbanna prefecture, 2006–2017.

### Multiple Regression Analysis

In Guangzhou city, the findings of multiple linear regression analysis suggested high correlations between Tmean and Tmin (*r* = 0.983), Tmean and Tmax (*r* = 0.953), Tmax and Tmin (*r* = 0.895), Tmean1 and Tmin1 (*r* = 0.968), Tmean1 and Tmax1 (*r* = 0.950), Tmax1 and Tmin1 (*r* = 0.865).

In addition, correlations also existed between DP and Hum (*r* = 0.661), DP and Tmin (*r* = 0.623), DP and CP (*r* = 0.754), as well as DP1 and CP1 (*r* = 0.633).

In Xishuangbanna prefecture, the results of multiple regression analysis showed high correlations between Tmean and Tmin (*r* = 0.949). In addition, correlations also existed between Tmean and Tmax (*r* = 0.783), DP and Tmin (*r* = 0.767), DP and CP (*r* = 0.755), and DP1 and CP1 (*r* = 0.645).

### Principal Component Analysis

Based on the above findings, multiple collinearities existed among some variables in both Guangzhou city and Xishuangbanna prefecture. Therefore, principal component analysis was adopted to avoid possible multiple collinearities. The eigenvalues, contribution variance and cumulative contribution of the variance of components in Guangzhou city and Xishuangbanna prefecture are shown in Table 2.

**Table 2:** The eigenvalues, contribution variance and cumulative contribution of variance of components in Guangzhou city and Xishuangbanna prefecture, 2006–2017.

**Research sites**	**Component**	**Variance**	**Contribution of variance (%)**	**Cumulative contribution of variance (%)**
Guangzhou city, Guangdong	1	4.577	35.211	35.211
	2	3.704	28.489	63.701
	3	1.258	9.676	73.376
	4	1.106	8.506	81.882
	5	0.812	6.243	88.125
	6	0.653	5.027	93.152
	7	0.341	2.623	95.775
	8	0.282	2.173	97.947
	9	0.166	1.273	99.22
	10	0.054	0.414	99.635
	11	0.04	0.310	99.945
	12	0.005	0.037	99.982
	13	0.002	0.018	100.00
Xishuangbanna prefecture, Yunnan	1	4.102	31.557	31.557
	2	3.025	23.270	54.827
	3	1.500	11.540	66.367
	4	1.272	9.788	76.155
	5	1.016	7.818	83.973
	6	0.753	5.793	89.765
	7	0.482	3.711	93.476
	8	0.318	2.445	95.921
	9	0.229	1.765	97.687
	10	0.191	1.473	99.159
	11	0.082	0.631	99.791
	12	0.021	0.159	99.950
	13	0.006	0.050	100.00

*1. Im1, the number of imported dengue cases lagged by 1 month; 2. BI, Breteau index; 3. Tmean, monthly average of temperature (°C); 4.Tmean1, monthly average of temperature lagged by 1 month (°C); 5. Hum, monthly average of relative humidity (%); 6.Tmin, monthly average of minimum temperature (°C); 7.Tmin1, monthly average of minimum temperature lagged by 1 month (°C); 8. Tmax, monthly average of maximum temperature (°C); 9. Tmax1, monthly average of maximum temperature lagged by 1 month (°C); 10. CP, cumulative precipitation; 11. CP1, cumulative precipitation lagged by 1 month; 12. DP, days of precipitation; 13. DP1, days of precipitation lagged by 1 month*.

### Principal Component Regression Analysis in Guangzhou City

After principal component analysis, multiple linear regression model was established using dengue cases as dependent variable and Z value as the independent variable.

PCR model parameter evaluation is shown in Table 3. It was found that the established model was statistically significant (*r* = 0.881, *F* = 11.899, *P* < 0.001). Results of *T* test revealed that the model constant, regression (REGR) factor score 1 for analysis 1, REGR factor score 4 for analysis 1, have the statistically significance (*P* = 0.034, 0.012 and < 0.001) (Table 4). The structure of established PCR using standardized scores of principal components in Guangzhou is as follows.

1. $\hat{Y}$ = 713.30-856.45 *Z*_1_+2103.97 *Z*_4_
  The structure of established linear regression model using original variable in Guangzhou is as follows.
2. $\hat{Y}$ = -14127.77+175.90 Im1+506.08 BI-32.06 Tmean+44.94 Tmean1-66.29 Tmin+38.22 Tmin1+139.10 Tmax-171.12 Tmax1+131.04 Hum+0.24 CP+1.91 CP1-6.74DP+130.12 DP1.

**Table 3:** Model parameter test in Guangzhou city, Guangdong province, 2006–2017.

**Model**	***R***	***R*^**2**^**	**Adjusted *R*^**2**^**	**Error of standard estimation**	***F***	***p***
1	0.881	0.776	0.725	2537.58	11.899	<0.001

**Table 4:** *T* test results of partial regression coefficient of the model in Guangzhou city, Guangdong province, 2006–2017.

**Model**		**Non standardized partial regression coefficient**		**Standardized partial regression coefficient**	***t***	***P***
		**Partial regression coefficient**	**Standard error**	**Partial regression coefficient**		
1	Constant	713.300	327.600		2.177	0.034
	REGR factor score 1 for analysis 1	−856.454	330.365	−0.256	−2.592	0.012
	REGR factor score 2 for analysis 1	−54.908	330.365	−0.016	−0.166	0.869
	REGR factor score 3 for analysis 1	−177.545	330.365	−0.053	−0.537	0.593
	REGR factor score 4 for analysis 1	2103.971	330.365	0.629	6.369	0.000

In Guangzhou, model revealed that the dengue occurrence was positively correlated with Im1, BI, Tmean1, Tmin1, Tmax, Hum, CP, CP1, DP1, and that was negatively correlated with Tmean, Tmin, Tmax1, and DP. The top three factors contributing to dengue were BI, Im1, and Tmax1.

### Principal Component Regression Analysis in Xishuangbanna Prefecture

Similarly, after principal component analysis, multiple linear regression model was established in with dengue cases as dependent variable and Z value as the independent variable.

Model parameter evaluation for Xishuangbanna is shown in Table 5. The established model was statistically significant (*R* = 0.898, *F* = 3.557 and *P* < 0.01). Results of *T* test showed that REGR factor score 2 for analysis 1, REGR factor score 4 for analysis 1, have the statistically significance (*P* = 0.014 and 0.015) (Table 6). The structure of established PCR using standardized scores of principal components in Xishuangbanna prefecture, Yunan province, is as follows.

(3) $\hat{Y}$ = 55.57+45.62 *Z*_2_+45.18 *Z*_4_
The structure of established linear regression model using original variable in Xishuangbanna prefecture, Yunan province, is as follows.
(4) $\hat{Y}$ = 3.40 Im1-2.52 BI-0.67 Tmean+2.00 Tmean1+0.60 Tmin+14.80 Tmin1+ 6.2 Tmax-1.19 Tmax1+5.32 Hum-0.07 CP+0.13 CP1-0.095 DP+2.80 DP1.

**Table 5:** Model parameter test in Xishuangbanna prefecture, Yunnan province, 2006–2017.

**Model**	***R***	***R*^**2**^**	**Adjusted R^**2**^**	**Error of standard estimation**	***F***	***p***
1	0.898	0.806	0.778	137.748	3.557	<0.01

**Table 6:** *T* test results of partial regression coefficient of the model in Xishuangbanna prefecture, Yunnan province, 2006–2017.

**Model**		**Non standardized partial regression coefficient**		**Standardized partial regression coefficient**	***t***	***P***
		**Partial regression coefficient**	**Standard error**	**Partial regression coefficient**		
1	Constant	55.567	17.783		3.125	0.003
	REGR factor score 1 for analysis 1	−31.033	17.933	−0.204	−1.730	0.089
	REGR factor score 2 for analysis 1	45.623	17.933	0.300	2.544	0.014
	REGR factor score 3 for analysis 1	6.636	17.933	0.044	0.370	0.713
	REGR factor score 4 for analysis 1	45.178	17.933	0.297	2.519	0.015
	REGR factor score 5 for analysis 1	24.295	17.933	0.160	1.355	0.181

In Xishuangbanna prefecture, the dengue occurrence was positively correlated with Im1, Tmean1, Tmin, Tmin1, Tmax, Hum, CP1, DP1, and was negatively correlated with BI, Tmean, Tmax1, CP, and DP. The top three factors contributing to dengue occurrence were Tmin1, Tmax, and Hum.

## Discussion and Conclusion

Guangzhou city (12, 35) and Xishuangbanna prefecture (17) are two representative regions of dengue outbreak with different dengue vectors during recent years in China (21). The actual number of dengue cases may be underestimated in the current notification system because of numbers of undetected cases with subclinical infections and atypical symptoms (36). Therefore, identifying the key factors influencing the occurrence of dengue in the two high-risk areas in this study is of great significance for the scientific control of dengue in the future.

At present, many studies focused on the influencing factors relevant to the local transmission, even outbreaks of dengue in representative regions of mainland China, including the two cities or regions in this study (7). Usually, dengue outbreaks are the consequence of the combination of many favorable conditions (6, 8). Unfortunately, some key factors were neglected in most previous studies, and the complex collinearity among the studied factors was also not well considered in these researches. Therefore, it is difficult to reveal the true contribution of different influencing factors. Furthermore, inconsistent conclusions existed in different reports due to different study periods and regions selected.

Based on the previous research, a total of 13 potential influencing factors, including climatic factors, indigenous and imported dengue cases, and mosquito larvae density (BI), were included and analyzed systematically using principal component regression analysis to avoid the possible multiple collinearity and confounding bias (33).

In Guangzhou, a region of dengue epidemic area dominated by *Aedes albopictus* (37), we observed multiple collinearity among variables. Specifically, high correlations existed among monthly maximum, minimum and mean temperatures, and these indicators lagged by 1 month, respectively with correlation coefficient higher than 0.85. In addition, correlations also existed among DP, and humidity, DP and Tmin, and DP and CP, DP1 and CP1, with the correlation higher than 0.60. Similar correlations existed for Xishuangbanna prefecture, where *Ae. aegypti* is the dominated vector for dengue transmission.

In this study, the established PCR model in Guangzhou revealed that the key contributing factors for the occurrence of dengue included BI (positive correlation), and Im1 (positive correlation). Dengue is transmitted by the biting of *Aedes* mosquito. Therefore, as one of a major indicators of mosquito density, BI is regarded as a promising and direct index for dengue early warning worldwide (38). As far as we know, the dengue case is the source of infection and transmission of dengue involving two incubation periods: an internal incubation period (3–15 days) of the dengue case and an external incubation period (3–15 days) in the body of *Aedes* mosquito. Furthermore, as an international metropolis, Guangzhou has a large number of floating populations from other domestic provinces and also foreign countries. Additionally, the major driving factor of Im1 identified in Guangzhou also confirmed that the risk of local outbreak caused by imported dengue case in this city is relatively high. This finding was in accordance with previous study focusing on Guangzhou city (34).

Temperature is regarded as one of the most important climatic factors for dengue transmission (39). Based on literature review, appropriate temperature could influence the reproduction of vector mosquitoes and dengue viruses, which subsequently impacts the dengue risk (40). Relative humidity can affect oviposition, egg hatching, dispersal range, feeding behaviors, and lifespan of *Aedes* mosquitoes. Our results in Xishuangbanna demonstrated that the top three factors driving dengue occurrences included Tmin1 (positive correlation), Tmax (positive correlation), and Hum (positive correlation). Xishuangbanna prefecture has a much smaller number of floating populations compared to Guangzhou. In particular, Xishuangbanna belongs to the tropical rain forest area, and the suitable meteorological conditions are more conducive to *Aedes* mosquito breeding, favoring the local outbreak of dengue. The findings were consistent with those in the previous investigation conducted in the border areas of Yunnan and Myanmar [BYM]) (7). Due to the relatively little evidence in Xishuangbanna prefecture of Yunnan, more future studies are warranted to further explore the key factors driving the local outbreak of dengue in this area.

Overall, our study observed that meteorological factors, especially Tmin1, Tmax, Hum, played key roles in dengue occurrence in Xishuangbanna, Yunnan while mosquito density (BI) and Im1 played important roles for dengue transmission in Guangzhou, Guangdong. For targeted dengue control in Guangzhou city, it is urgent to pay close attention to the dengue epidemic both at home and abroad, track the imported dengue cases timely, and reduce adult mosquito density relying on source reduction of *Aedes* mosquitoes. As for dengue control in Xishuangbanna, it is necessary to include some key meteorological factors when the implementation of dengue early warning and precise control. Furthermore, the difference of dengue determinants in different provinces could be taken into account when formulating tailored dengue precise control and prevention strategy, response mechanism in representative areas of China in the future.

Some limitations should be noticed. First, the impact factors of dengue local transmission and outbreaks are complicated. We have tried our best to include as many risk factors as possible. However, other factors, such as public health control programs (30), new dengue virus strain invasion (41, 42), emerging insecticide resistance in dengue vectors (43, 44), El Nino Southern Oscillation (28), changes of land use and surface water (2), key areas in a city such as “urban villages (UVs)” (3), social media surveillance data (45), air travel data (46), human behavior, other socio-economic indicators (47), are also important for the dengue occurrence and need to be well discussed in future studies. In addition, the exact number of cases of dengue may be underestimated (36) due to the phenomenon of latent infection and mild cases of dengue, which were not included in this study.

## Conclusions

Climatic factors and mosquito density are the key drivers on dengue occurrence in representative high-risk dengue areas in China. Meteorological factors such as monthly average of minimum temperature lagged by 1 month, monthly average of maximum temperature and relative humidity, have stronger impacts on dengue occurrence in Xishuangbanna prefecture of Yunnan, while *Aedes* larvae density and the number of imported dengue cases lagged by 1 month have more profound impacts on dengue occurrence in Guangzhou city. The findings may provide scientific evidence for the development of early warning and targeted dengue control strategies and measures in China.

## Data Availability Statement

The data analyzed in this study is subject to the following licenses/restrictions: Some data could be available based on China CDC's regulations. Requests to access these datasets should be directed to liuxiaobo@icdc.cn.

## Ethics Statement

This study was approved by the Ethics Committee of National Institute for Communicable Disease Control and Prevention, China CDC. No human or animal samples were included in the current study.

## Author Contributions

XL planned the project and wrote the paper. XL, QL, and YG conducted the field survey. XL, KL, YY, HW, SY, DR, NZ, and JY contributed to data analysis. All authors read and approved the final manuscript.

## Conflict of Interest

The authors declare that the research was conducted in the absence of any commercial or financial relationships that could be construed as a potential conflict of interest.
